# Supported Porous Nanostructures Developed by Plasma Processing of Metal Phthalocyanines and Porphyrins

**DOI:** 10.3389/fchem.2020.00520

**Published:** 2020-06-17

**Authors:** Jose M. Obrero, Alejandro N. Filippin, Maria Alcaire, Juan R. Sanchez-Valencia, Martin Jacob, Constantin Matei, Francisco J. Aparicio, Manuel Macias-Montero, Teresa C. Rojas, Juan P. Espinos, Zineb Saghi, Angel Barranco, Ana Borras

**Affiliations:** ^1^Nanotechnology on Surfaces and Plasma Laboratory, Materials Science Institute of Seville (ICMS, CSIC-US), Seville, Spain; ^2^Departamento de Física Atómica, Molecular y Nuclear, Universidad de Sevilla, Seville, Spain; ^3^Université Grenoble Alpes, CEA, LETI, Grenoble, France; ^4^Instituto de Óptica Daza Baldés (CSIC), Madrid, Spain

**Keywords:** supported porous nanomaterials, porous thin films, plasma deposition, nanowires, nanotubes, phthalocyanine, 3D characterization, electron tomography

## Abstract

The large area scalable fabrication of supported porous metal and metal oxide nanomaterials is acknowledged as one of the greatest challenges for their eventual implementation in on-device applications. In this work, we will present a comprehensive revision and the latest results regarding the pioneering use of commercially available metal phthalocyanines and porphyrins as solid precursors for the plasma-assisted deposition of porous metal and metal oxide films and three-dimensional nanostructures (hierarchical nanowires and nanotubes). The most advanced features of this method relay on its ample general character from the point of view of the porous material composition and microstructure, mild deposition and processing temperature and energy constrictions and, finally, its straightforward compatibility with the direct deposition of the porous nanomaterials on processable substrates and device-architectures. Thus, taking advantage of the variety in the composition of commercially available metal porphyrins and phthalocyanines, we present the development of metal and metal oxides layers including Pt, CuO, Fe_2_O_3_, TiO_2_, and ZnO with morphologies ranging from nanoparticles to nanocolumnar films. In addition, we combine this method with the fabrication by low-pressure vapor transport of single-crystalline organic nanowires for the formation of hierarchical hybrid organic@metal/metal-oxide and @metal/metal-oxide nanotubes. We carry out a thorough characterization of the films and nanowires using SEM, TEM, FIB 3D, and electron tomography. The latest two techniques are revealed as critical for the elucidation of the inner porosity of the layers.

## Introduction

**Scheme 1:**
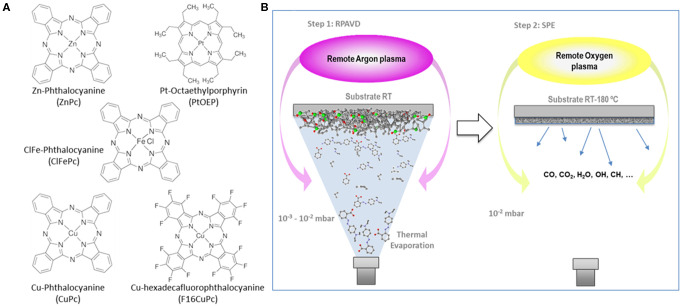
**(A)** Chemical structure of some of the metal phthalocyanines and porphyrins tested in this article. **(B)** Schematic representation of the remote plasma-assisted vacuum deposition process (RPAVD) and the soft plasma etching process (SPE).

The synthesis of nanostructured porous metal and metal oxide nanomaterials has become imperative to the development of functional and catalytic applications because of their tunable physical and chemical properties, high surface area and advantageous use as the host material in the novel hybrid and heterostructured systems (Morris and Wheatley, 2008; Shiju and Guliants, 2009; Kim and Nair, 2013; Zhu et al., 2015; Wang et al., 2017; Jin and Maduraiveeran, 2019). Traditionally, much more attention has been invested in the colloidal synthesis of porous materials in the form of powders and metallorganic frameworks than in the synthesis of supported nanoporous films and low dimensional nanomaterials (Morris and Wheatley, 2008; Shiju and Guliants, 2009; Dhakshinamoorthy and Garcia, 2012; Kim and Nair, 2013; Zhu et al., 2015; Zhao et al., 2016; Wang et al., 2017; Jin and Maduraiveeran, 2019). However, during the last decade, the interest in the controlled deposition of metal and metal oxide (MOs) porous nanosystems has been fostered by the advanced and emergent applications in areas, such as photonics, photovoltaics, energy harvesting and storage, smart surfaces and nanosensors (Romero-Gómez et al., 2010; Sánchez-Valencia et al., 2010; Cerofolini et al., 2011; Sun et al., 2012; Zhang et al., 2012; Dave and Malpani, 2014; Wu et al., 2014a,b; Barranco et al., 2016; Sk et al., 2016; Ferrando-Villalba et al., 2018; Ramirez-Gutierrez et al., 2019; Luo et al., 2020). A common feature of these applications is the need for the on-base growth of the porous nanomaterial from a processable substrate, such as transparent conducting oxides (TCOs), or on-device architecture, such as interdigitated electrodes (Romero-Gómez et al., 2010; Sánchez-Valencia et al., 2010; Cerofolini et al., 2011; Sun et al., 2012; Zhang et al., 2012; Dave and Malpani, 2014; Wu et al., 2014a,b; Barranco et al., 2016; Sk et al., 2016; Ferrando-Villalba et al., 2018; Ramirez-Gutierrez et al., 2019; Luo et al., 2020). In consequence, the number of publications devoted to the tunable deposition of metal and metal oxide porous systems including chemical solution methods, electrodeposition or electrospinning, and vacuum phase as physical vapor deposition and chemical vapor deposition has enormously increased (Hodes, 2007; Jerónimo et al., 2007; Romero-Gómez et al., 2010; Sánchez-Valencia et al., 2010; Cerofolini et al., 2011; Kim and Rothschild, 2011; Sun et al., 2012; Zhang et al., 2012; Pal and Bhaumik, 2013; Dave and Malpani, 2014; Lee and Park, 2014; Sun and Xu, 2014; Wu et al., 2014a,b; Malgras et al., 2015; Barranco et al., 2016; Sk et al., 2016; Xue et al., 2017; Ferrando-Villalba et al., 2018; Liu et al., 2018; Coll and Napari, 2019; Coll et al., 2019; Ramirez-Gutierrez et al., 2019; Luo et al., 2020; Siebert et al., 2020). In the case of vacuum phase approaches, the methodologies previously developed for the synthesis of highly compact films, such as thermal, electron-beam or ion-assisted evaporation, magnetron sputtering, atomic layer deposition (ALD), and plasma enhanced chemical vapor deposition (PECVD) have been thoroughly modified and expanded to produce microporous and mesoporous layers (Romero-Gómez et al., 2010; Sánchez-Valencia et al., 2010; Borras et al., 2012; Barranco et al., 2016; Coll and Napari, 2019; Coll et al., 2019). Strategies, such as deposition in glancing angle conditions (Barranco et al., 2016) or the use of sacrificial soft and hard templates (Pal and Bhaumik, 2013; Lee and Park, 2014; Sun and Xu, 2014; Malgras et al., 2015) have allowed the fabrication of metal and metal oxide layers endowed with under-design porosity, microstructure and structure as well as with strict control on the chemical bulk and surface composition, and functionalization. Thus, the advantages of the use of vacuum deposition methods relay on both, the strict control on the chemical composition of the resulting material, and the direct large scale and industrial implementation of these techniques. Vacuum phase deposition methods are therefore applied in many fields because of their low byproducts yield, solventless nature, straightforward automatization and compatibility with multi-step deposition procedures and roll-to-roll fabrication (Borras et al., 2012; Barranco et al., 2016; Coll and Napari, 2019; Coll et al., 2019; Tian et al., 2019). In the concrete case of plasma-based approaches, such as PECVD, Plasma assisted ALD or magnetron sputtering, it is worth to mention that the energy balance is as well positive. Hence, the substrate temperatures to obtain high-quality materials, including crystalline systems and low dimensional nanostructures, remain within a mild range (Barranco et al., 2016; Joseph et al., 2018; Brandenburg et al., 2019; Chiang et al., 2019; Ostrikov, 2019; Tian et al., 2019; Weltmann et al., 2019). This makes the plasma-assisted methods very attractive for the development of porous layers on temperature sensitive substrates and in applications intended for an efficient payback period, which is the case in microelectronics, photovoltaic, optic, and automotive industries, among others (Joseph et al., 2018; Brandenburg et al., 2019; Chiang et al., 2019; Ostrikov, 2019; Tian et al., 2019; Weltmann et al., 2019). In this article, we will summarize our latest results regarding the development of a hybrid thermal evaporation, plasma deposition and etching approach for the formation of metal and metal oxide porous layers using phthalocyanine and porphyrin molecules as solid metal precursors (see Scheme 1A) for the chemical structure of some of the molecules tested in this article). This procedure evolves from the Remote Plasma Assisted Vacuum Deposition (RPAVD) method previously intended for the fabrication of organic (polymer-like) layers from functional organic molecules (see Scheme 1B) (Barranco and Groening, 2006; Aparicio et al., 2011, 2012, 2014, 2016; Idígoras et al., 2018; Alcaire et al., 2019a). In addition to the already mentioned features of the vacuum and plasma deposition approaches, the use of metal phthalocyanines and porphyrins as solid precursors may surpass traditionally applied organometallic liquids and vapors in several aspects, among them: (i) they can be straightforwardly handled in vacuum due to their low sublimation temperatures and high stability, (ii) their use is harmless in comparison with standard organometallic precursors (low toxicity, non-inflammable solid material), (iii) these are already commercially available molecules with relatively facile production in high volume, (iv) there is an ample variety regarding the metal cations with a multitude of available transition metals; (v) fabrication of multilayers or doped nanoporous oxides are achievable by sequential or simultaneous processing of porphyrins and phthalocyanines with different metal cations and or peripheric ligands and finally, (vi) this method is extensible to other sublimable functional metal complexes (Alcaire et al., 2011, 2016; Filippin et al., 2017a).

An additional advantage of the use of these molecules deals with their application as building blocks for the formation of low dimensional organic nanostructures through self-assembly. The assembly mechanism is driven by supramolecular interactions, such as van der Waals forces (Borras et al., 2008, 2010; Briseno et al., 2008a,b; Zhang et al., 2014; Zong et al., 2019; Mirabito et al., 2020). These molecules possess an extensive network of delocalized electrons, the so-called π-electrons, which allow them to undergo aromatic-aromatic interactions and stack one over the other through π-stacking. The self-assembly in highly π-conjugated planar systems is mainly dominated by these π-π interactions, but it is largely affected by the type of substituents and central metal cation which can lead to different nanostructured motifs, such as tubes, rods, sheets, nanowires, etc. In this article, we will profit from this feature to go a step forward and fabricate supported porous core@shell nanowires and nanotubes. With this aim, we will extend a soft-template methodology based on the use of single-crystalline organic nanowires as supported one-dimensional and three-dimensional templates.

The characterization of these porous systems is not an easy task since the traditional techniques as N_2_ isotherms fail in the elucidation of pore-size distributions. This is mainly due to the fact that the usual amount of material formed for on-surface or on-device purposes does not reach the minimum weight limit for these methods. Other approaches as optical isotherms using UV-Vis or ellipsometry spectroscopies and room temperature isotherms provided by a quartz crystal microbalance (QCM) have been exploited during the last years (Borras et al., 2007b, 2012). However, these approaches require high optical quality, which hinders their application in scattering media, as 3D nanowires, or their combination with additional testing as Rutherford Backscattering Spectroscopy (RBS) to determine the material density (Borrás et al., 2006). Destructive and non-destructive investigation of the 3D porosity and pore size distribution in thin films and low dimensional nanostructures can be conducted using different forms of tomography. Atom probe tomography (APT) provides 3D information about the structure and composition of materials with sub-nanometer resolution. For nanoporous materials to be analyzed by APT, it is essential to fill the pores in order to obtain compact materials. This was successfully achieved by electron beam induced deposition for nanoporous gold with pore diameter of 50 nm (Pfeiffer et al., 2015), and by electrochemical filling for nanoporous silicon with pore diameter of 10 nm (Mouton et al., 2017). Electron tomography (ET) utilizes a transmission electron microscope (TEM) for the acquisition of a set of projections at different tilt angles, over as wide a tilt range as possible. The 3D object is then reconstructed after alignment of the tilt series, with a resolution in the nanometer range. ET has played a critical role in the study of catalyst materials (e.g., Friedrich et al., 2009), porous thin films (e.g., Biermans et al., 2010; Mula et al., 2017), and nanowires (e.g., Ferrando-Villalba et al., 2018), but has a field of view limited to few hundreds of nanometers. For larger volumes, focused ion beam (FIB) milling combined with scanning electron microscopy (SEM) provides 3D reconstructions with a voxel size of ~10 nm, although 3 nm is achievable, as reported in Cantoni et al. (2010), and a field of view of few microns. Applied to porous structures, such as solid oxide fuel cells (Iwai et al., 2010; Sabharwal et al., 2017), this technique (FIB-3D) gives invaluable information about the microstructural properties of the materials, and the volumes generated can serve as models for the estimation of transport properties. FIB-3D, however, requires the impregnation of the pores with epoxy resin, which can induce changes in the structure, as suggested in Sabharwal et al. (2017). For similar fields of view, nanoscale X-ray computerized tomography (nano-CT) produces 3D reconstructions at ~50 nm spatial resolution and in a non-destructive way, making it interesting for *in-situ* studies. Compared to FIB-3D, nano-CT does not require epoxy impregnation, but the acquisition time is longer. A more in-depth comparison of the two techniques can be found in Wargo et al. (2013). In this work, we used FIB-3D for the analysis of a supported nanoporous oxide thin film and electron tomography for the 3D reconstruction of an isolated TiO_2_ porous nanotube. These 3D characterizations were complemented by classical SEM (planar and cross-section views), and 2D TEM, STEM, and EDX.

## Results and Discussion

### Remote Plasma-Assisted Vacuum Deposition of Porous Metal Oxide Thin Films

**Figure 1:**
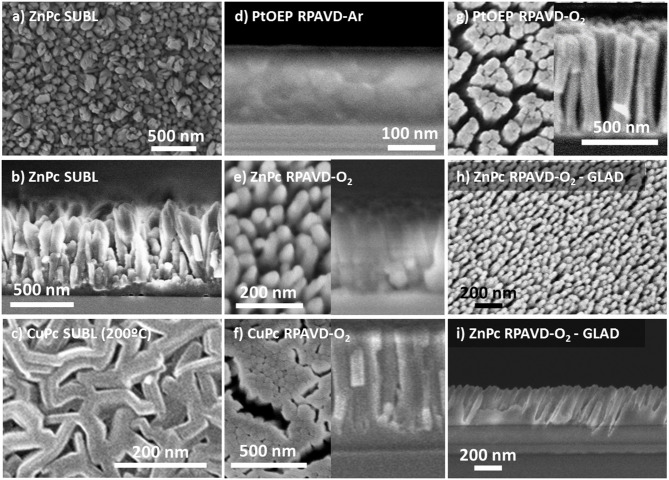
Planar view and cross-section SEM micrographs of representative samples as labeled (see also Table 1 at the Experimental Section): **(a,b)** planar and cross-sections corresponding to the direct sublimation of ZnPc molecules with the substrate at room temperature; **(c)** Planar view of a CuPc sublimated sample with the substrate temperature at 200°C; **(d)** Cross-section of a sample Remote plasma assisted vacuum deposited sample of PtOEP using Ar plasma; **(e–g)** planar (left) and cross-section (right) views of ZnPc, CuPc, and PtOEP molecules deposited by RPAVD-O_2_; **(h,i)** Planar and cross-section views of the ZnPc deposition at glancing angles supported by remote oxygen plasma.

The RPAVD methodology has been developed in recent years for the formation of multifunctional nanocomposite organic films showing an enhanced performance in applications, such as UV and gas sensors, lasing media, hydrophobic coatings, and encapsulation of fragile matter (Barranco and Groening, 2006; Aparicio et al., 2011, 2012, 2014, 2016; Idígoras et al., 2018; Alcaire et al., 2019a). In standard operation conditions, the RPAVD method consists of the thermal evaporation of the precursor molecules in the afterglow region of a microwave plasma supported by electron cyclotron resonance (ECR), i.e., in a downstream configuration (see Scheme 1B). The substrates are placed facing the evaporation source and back to a microwave plasma discharge. The deposition is carried out with the substrates at room temperature and in the presence of Ar as plasma gas. In our previous articles, we have focused on the application of this method to the development of layers fabricated with organic functional materials as flavonols, perylenes, rhodamines, and adamantane (Barranco and Groening, 2006; Aparicio et al., 2011, 2012, 2014, 2016; Idígoras et al., 2018; Alcaire et al., 2019a). Herein, we include two modifications, on one hand, the use of metal conjugated molecules, metal porphyrins and phthalocyanines, and on the other, the use of oxygen-rich plasmas and plasma etching post-treatments. The use of metallorganic molecules will pave the way for the development of metal and metal oxide layers, meanwhile, the application of oxygen plasma procedures will allow the formation of columnar oxide layers. Figure 1 gathers different cross-section and normal view SEM micrographs of representative samples prepared by this method (Table 1 in the Experimental Section includes the detailed experimental conditions). Samples labeled as SUBL were fabricated by the direct sublimation of the molecules without plasma activation (panels a-c), RPAVD-Ar were deposited applying a remote Ar plasma (d) and RPAVD-O_2_ combining Ar and O_2_ as plasma gases (e-i). All the experiments were carried out with the substrates at room temperature except the example in panel (c). Parameters, such as the deposition rate, thickness, substrate geometry, and plasma power were tuned to show an overview of the versatility of the method. Thicknesses and growth rates were monitored by a QCM and settled in the range between 200 and 600 nm and 0.4 and 0.8 Å/s, respectively. The microstructures of the samples both in cross-section and planar view are obviously different. The thermal evaporation conditions produce a polycrystalline conformation (Borras et al., 2010) with randomly oriented grains formed by the metallic complex molecules. The Ar plasma-assisted deposition generates smooth samples with almost inappreciable features at the nanoscale. This is in good agreement with previous results regarding the RPAVD deposition of organic functional molecules showing root mean square roughness in the order of 0.5 nm (in 1 × 1 μm) for films several hundred nanometers thick (Idígoras et al., 2018; Alcaire et al., 2019a). The RPAVD-O_2_ conditions yield in all the cases a nanocolumnar morphology. These columns present narrow diameter and length size distributions, giving rise to highly homogeneous samples with well-defined features. The conditions selected in Figure 1 produce samples with column widths in the order of 65 nm for both CuPc and PtOEP precursors, panels (f) and (g), correspondently. The columns present homogeneous thicknesses and widths from top to bottom. However, it is important to address that the nanocolumnar formation is preceded by a continuous layer. The thickness of this layer ranges from 75 (g) to 130 nm (f). This result might be in concordance with previous reports in the literature about the plasma enhanced chemical vapor deposition (PECVD) of nanocolumnar thin films (Borras et al., 2007a,b, 2012). These articles demonstrate that the eventual formation of the columns depends on both the shadowing effects (also depending on the sticking coefficient of the ad-species) and the roughness of the surface, in the way that a minimum roughness threshold is required to develop the initial shadowing mechanism responsible for the growth of this type of morphology (Coll et al., 2019). Higher sticking coefficients are indeed related to the application of O_2_ plasmas where the ad-species are easily oxidized at the surface of the growing films in comparison with Ar plasmas, usually determined by a pronounced diffusion-enhanced mechanism (Borras et al., 2007b). This also explains that the intermediate film is thinner for higher power plasmas (600 W, panel g) than for the lower (300 W, panel f). It is also worth mentioning that in our particular case, the self-shadowing effects are barely affecting the growth of the columns since they present a homogeneous diameter along their length and not the characteristical thicker tips (Borras et al., 2007a,b). It is important to stress herein that the exploitation of the shadowing mechanism to generate under design nanocolumnar layers had been marked during the last two decades by the leading role of the glancing angle fabrication methods mostly based on physical vapor deposition (PVD) approaches, as thermal and e-beam assisted deposition and magnetron sputtering. In fact, very few reports have appeared regarding the glancing angle deposition by CVD or PECVD methods (Barranco et al., 2016). In PVD glancing angle u oblique angle depositions (GLAD or OAD), the directionality of the precursor vapor is well-defined with respect to the growing surface and the simple tilting or rotation of the substrates with respect to a punctual source of precursor provide a plethora of possible morphologies, from vertical to tilted columns, including zig-zag and sculptured samples for organic, metal, metal oxide hybrid and heterostructured composition, amorphous and crystalline microstructures (Barranco et al., 2016). Thus, this approach has been successfully applied in fields defined by on-surface or on-devices strategies like optics, photonics, microfluidics, photovoltaics, magnetism and nanosensors, and the relationship between microstructure, porosity and enhanced properties has been thoroughly analyzed. In this article, we present the results of the first set of exploratory experiments combining the RPAVD-O_2_ conditions with the tilting of the substrate with respect to the precursor evaporation sources (Figures 1h,i). This sample was deposited under the very same conditions (0.4 A/s, 300 W, O_2_ plasma) as sample in panel (e) but with the substrate forming an angle ß ~ 80° with respect to the evaporation flux and under reduced O_2_ pressure, i.e., 1 × 10^−2^ compared to 3 × 10^−2^ mbar (see Experimental Section). The cross-section image depicts the characteristic tilted nanocolumnar formation of the samples deposited under glancing angle conditions, with the columns forming an angle α ~ 23 ± 2° (Barranco et al., 2016) The width of the nanocolumns is thinner for the RPAVD-O_2_-GLAD (34 ± 5 nm) than for the equivalent RPAVD-O_2_ conditions (52 ± 5 nm). This image also shows the initial granular region at the interface with the substrate which under these conditions reaches up to 200 nm. The normal view image corroborates the tilted alignment of the sample but does not present the budling effects (i.e., the agglomeration of the tips of the columns in a determined direction) often presented by GLAD layers (Barranco et al., 2016). Although already mentioned, these are preliminary experiments that pave the way for an alternative plasma-based glancing angle deposition approach.

**Table 1:** Experimental conditions selected for the synthesis of hybrid and metal oxide layers by RPAVD.

**Sample**	**Gas plasma**	**Deposition rate [Å/s]**	**Power [W]**	**Pressure [mbar]**
ZnPc RPAVD-O_2_	O_2_	0.4	300	3·10^−2^
ZnPc RPAVD-O_2_-GLAD	O_2_	0.4	300	1·10^−3^
CuPc RPAVD-O_2_	O_2_	0.4	300	3·10^−2^
F_16_CuPc RPAVD-O_2_	O_2_	0.4	150	3·10^−2^
PtOEP-RPAVD-Ar	Ar	0.4	300	3·10^−2^
PtOEP RPAVD-O_2_	Ar/O_2_	0.4	600	3·10^−2^
ClFePc RPAVD-O_2_	O_2_	0.4	600	3·10^−2^

**Figure 2:**
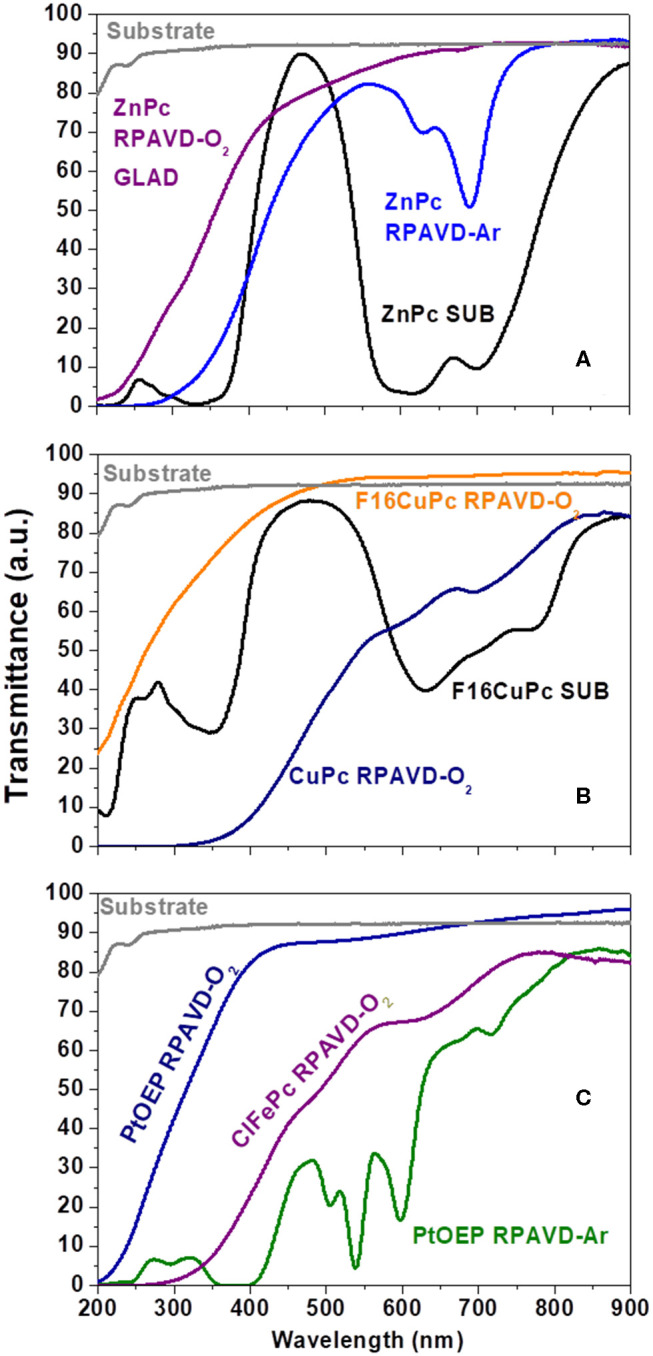
**(A-C)** UV-Vis Transmittance spectra comparing samples prepared under different experimental conditions as labeled.

**Figure 3:**
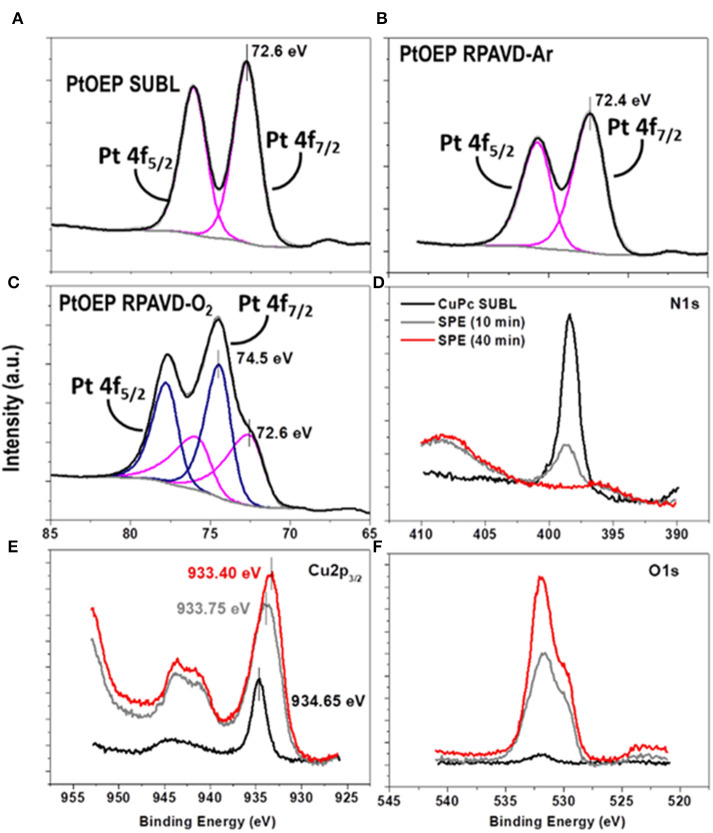
Pt 4f peaks obtained by XPS of the PtOEP samples deposited under direct sublimation **(A)**, RPAVD-Ar **(B)** and RPAVD-O2 **(C)**. Evolution of the N 1 s **(D)**, Cu 2p **(E)** and O 1s **(F)** peaks of the CuPc-SUBL sample after soft-plasma etching treatments of different durations.

It is well-known that the RPAVD-Ar procedure provides the formation of nanocomposite layers where the organic molecules appear embedded in a polymeric matrix consisting of the molecular fragments formed after the interaction of the precursor molecules with the plasma species (Barranco and Groening, 2006; Aparicio et al., 2011, 2012, 2014, 2016; Idígoras et al., 2018; Alcaire et al., 2019a). This striking feature has allowed the fabrication of solid, non-soluble and thermally stable layers with tunable properties from molecular functions to plasma-polymers. Figures 2, 3 and Table S1 (see also Alcaire et al., 2011, 2016; Filippin et al., 2017a), gather the UV-Vis transmittance spectra and X-Ray Photoelectron Spectroscopy (XPS) results aiming for the elucidation of the chemical composition of the samples, comparing direct sublimation, RPADVD-Ar, and RPAVD-O_2_ conditions. The transmittance spectra of the sublimated phthalocyanine samples in Figures 2A,B resemble those of the molecules (ZnPc and F16CuPc, respectively) with the characteristic Q and Soret absorption bands in the 600–800 and 300–400 nm correspondently. XPS results also indicate that the sublimated samples present the stoichiometry equivalent to the molecules (see Figure 3 and Table S1). On the other hand, in good agreement with previous results of the application of RPAVD-Ar, the spectra of the RPAVD-Ar samples contains bands corresponding to integer embedded molecules along with small moieties formed after partial plasma fragmentation of the molecule. This is also noticeable in the XPS results: for example, the spectrum of Pt 4f for the sample prepared under such conditions appears at binding energies corresponding to Pt into the PtOEP molecule (Filippin et al., 2017a) (see Figure 3B and Table S1). These integer molecules are embedded in the crosslinked matrix formed by fragmented molecules. On the other hand, the RPAVD-O_2_ conditions lead to a different situation: samples prepared at normal incidence angle are transparent in the visible range with the difference in the absorption edge and transparency depending on the molecule and the sample thickness (see also Filippin et al., 2017a). The deconvolution of the Pt 4f peaks in Figure 3C is compatible with the presence of Pt coming from the PtOEP molecules as well that in PtO and PtO_2_. This result indicates that the RPAVD-O_2_ method yields, in fact, hybrid layers containing integer molecules, oxidized fragments, and crosslinked molecular fragments. Finally, the samples prepared under RPAVD-O_2_ GLAD conditions present in addition slight absorption bands corresponding with the original molecule. This might indicate the weak oxidation of the molecules under such experimental parameters and it is in good agreement with the presence of thick continuous layers at the interface of the substrates previous to the formation of tilted nanocolumns.

**Figure 4:**
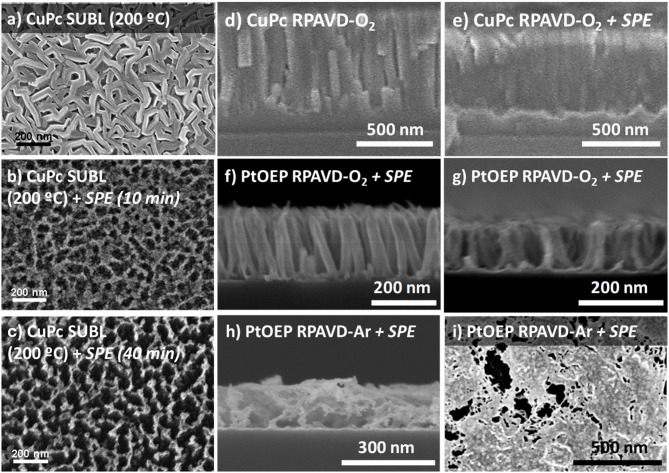
**(a-i)** Planar view and cross-section SEM micrographs representative of the soft-plasma etching results as labeled.

The ultimate conversion into metal or metal oxides of the hybrid layers can be achieved by post-processing with soft plasma etching (SPE) in the same reactor (Alcaire et al., 2011, 2016; Filippin et al., 2017a) (see Scheme 1B) under different combinations of oxygen and argon gases in the plasma and for temperatures ranging from RT to 180°C as gathered in Figure 4. Sublimated and RPAVD-O_2_ samples retain their columnar microstructure after the SPE treatments, which in addition produce three visible effects on the nanostructures: the aggregation of the nanocolumns leading to an increase of overall sample porosity as the columns tend to form bundles, reduction in the thickness when compared with the as-grown layers and rise of the surface roughness of such columns due to the formation of metal nanoparticles (Figures 4a–g). The increment in the temperature of the substrates and plasma etching time increases the size of the bundles and the distance between them (see panels b-c and Filippin et al., 2017a). PtOEP samples deposited under Ar plasma (RPAVD-Ar in Figures 4h,i), that originally consist or continuous and rather compact layers, experience a different transformation after SPE treatment: the microstructure is converted into a porous interconnected network.

**Figure 5:**
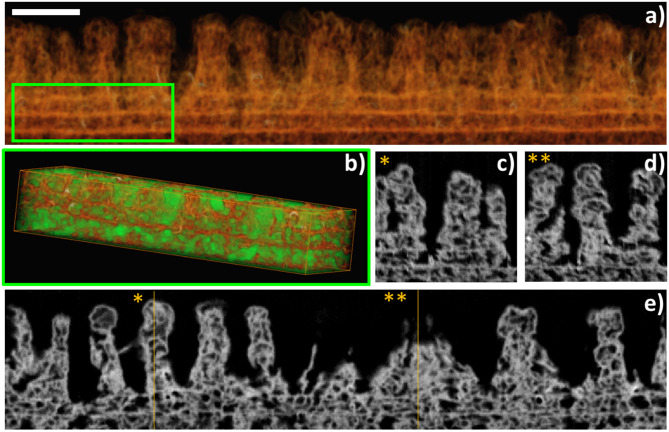
FIB-3D of a sample fabricated using RPAVD-O_2_ and soft plasma etching of ClTiPc. **(a)** Voxel rendering of the 3D reconstruction. **(b)** A subvolume was selected [inset in **(a)**] for the segmentation of the pores (displayed in green) and the porosity estimation. (**e**) is an xy slice through the volume. **(c,d)** are xz slices at the y positions * and ** indicated in **(e)**. Scalebar: 500 nm.

FIB-3D was performed on a sample fabricated by a similar process using ClTiPc as precursor, after epoxy impregnation and carbon coating (Figure 5). The field of view chosen for this experiment was 6 × 1.5 × 4 μm^3^, with a pixel size of 4 nm. Figure 5 shows the voxel rendering of a subvolume and selected slices through the reconstruction. This reconstruction provides an insight view of the highly porous systems generated after a complete post-treatment with pores showing a broad size distribution and diameters in the range between 150 and 10 nm in both the continuous and columnar counterpart of the samples. The porosity estimated for the bottom layer is ca. 56%. It is important to stress herein that this value might be underestimated since the pixel size is in the limit of resolution of the smaller pores, as we will discuss in the second section. The surface chemical composition of the as-grown samples and after several post-treatments was evaluated by means of *ex situ* XPS (see Figures 3D–F and Table S1). Auger parameters estimated for the CuPc as-grown and post-treated samples correspond to α (Cu) = 1850.85 eV (SUBL), 1851.05 eV (SPE 10 min), and 1851.00 eV (SPE 40 min). The last two values are compatible with the formation of CuO with small particle size (Espinos et al., 2002). In brief, the surface concentration of carbon and nitrogen decreases, and the oxygen arises, for ZnPc, CuPc, F_16_CuPc, ClTiPc, ClFePc, and PtOEP thin films after the soft-plasma etching post-treatment compared with the as-grown ones as expected. The amount of oxygen also has a strong dependence on the oxide stoichiometry and the amount of substrate [Si(100)] uncovered and also oxidized under the plasma treatment. In all the cases, we have achieved the complete conversion to the corresponding metal oxide after soft-plasma etching treatments of different durations and temperatures below 180°C. Table S1 probe that ZnO, TiO_2_, CuO, and Fe_2_O_3_ oxides can be eventually formed. In the particular case of PtOEP, it is also possible to form metal particles and layers after further annealing under Ar and H_2_ atmosphere (Filippin et al., 2017a).

### Development of Hybrid, Metal, and Metal Oxide Core@Shell Nanowires and Nanotubes

**Scheme 2:**
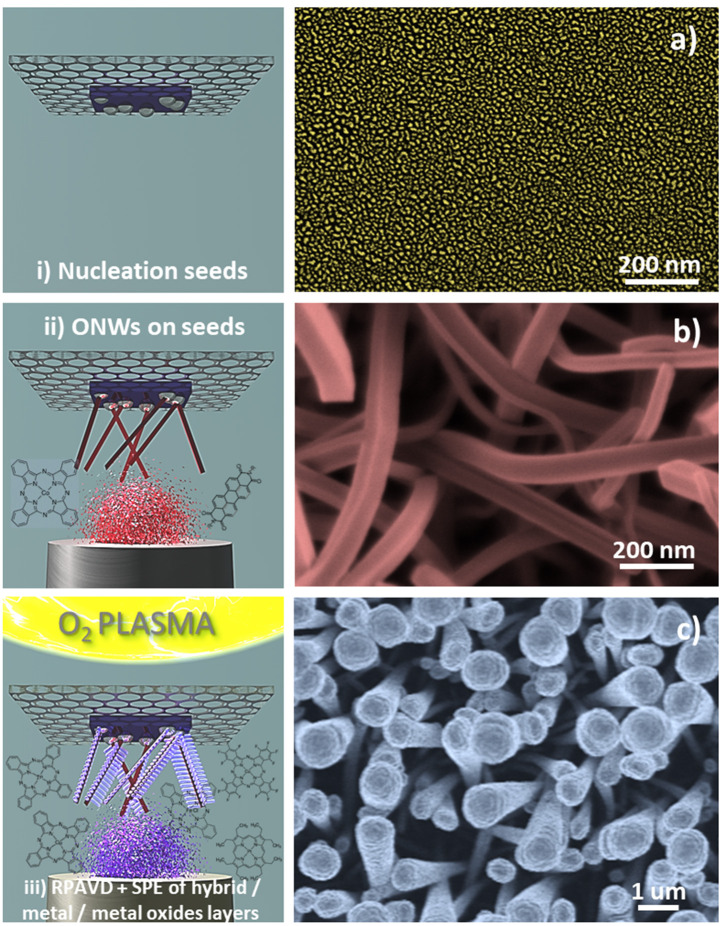
Steps for the formation process of the hierarchical core@shell nanostructures (left) and SEM micrographs characteristic of every step (right): **(i)** Deposition of metal nanoparticles or oxide thin film as nucleation centres. The image correspond to silver nanoparticles deposited on Si (100) by sputtering **(a)** (Filippin et al., 2019); **(ii)** Vapor transport deposition in vacuum of single-crystal organic nanowires (phthalocyanines, porphyrins, and perylenes), in the example H_2_Pc nanowires **(b)**; and **(iii)** Formation of the shell: Growth by remote oxygen plasma assisted-vacuum deposition at RT on the ONWs and posterior soft-plasma etching. The top view SEM image correspond to CuPc RPAVD-O_2_ deposited on H_2_Pc nanowires **(c)**.

**Figure 6:**
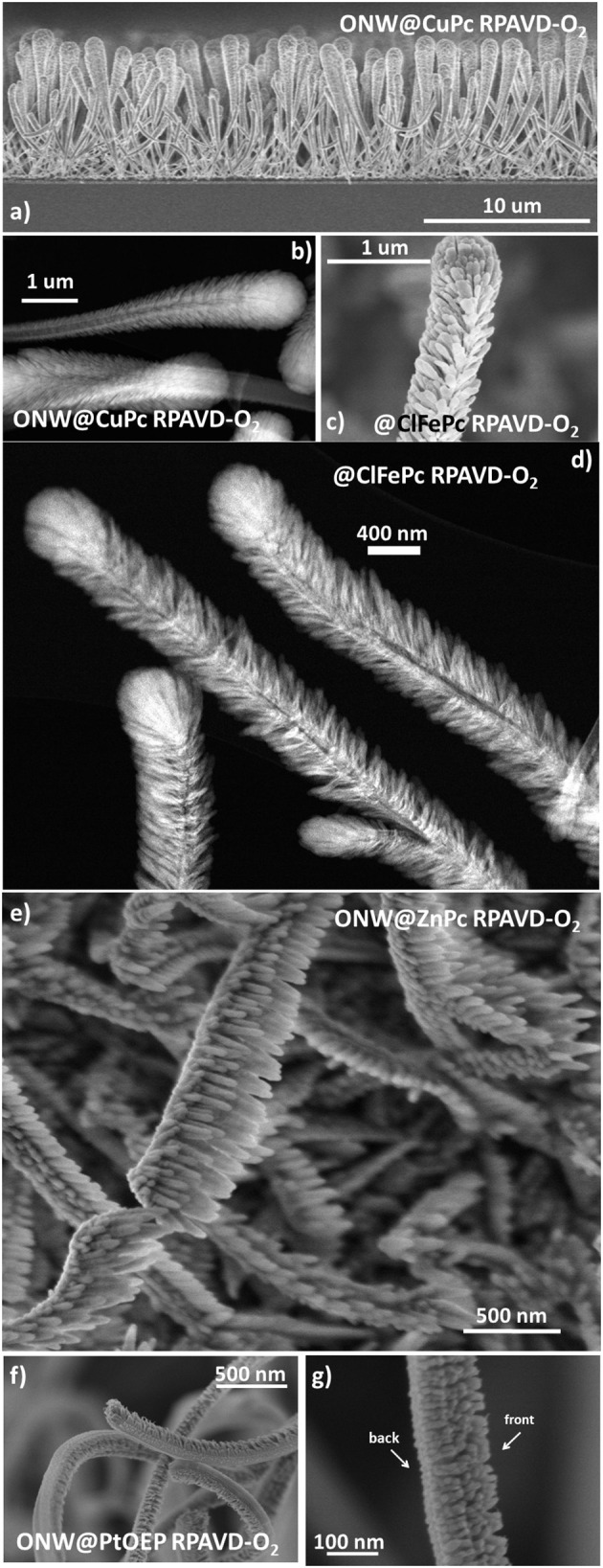
SEM **(a,c,e–g)** and HAADF-STEM **(b,d)** micrographs of core@shell nanowires formed by ONWs of H_2_Pc **(a,b)** and MePTCDI **(e,f)**. The micrograph in **(d)** shows the formation of ClFePc nanotubes after removing the H_2_Pc cores.

**Figure 7:**
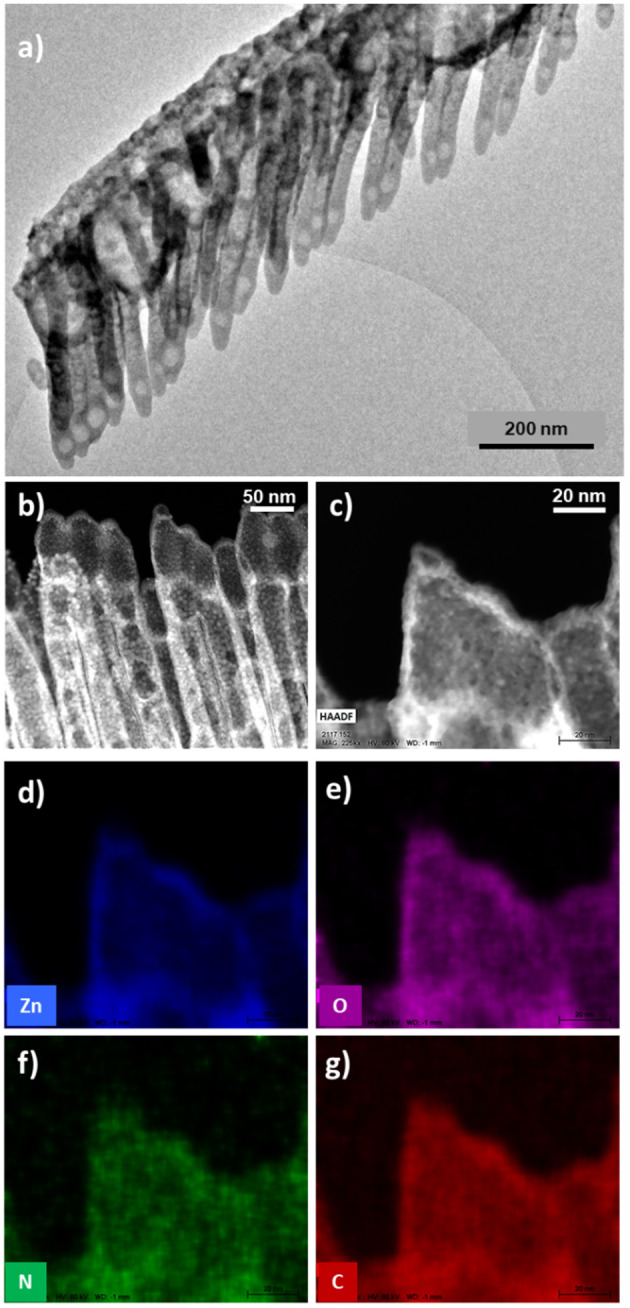
TEM **(a)**, HAADF-STEM **(b-c)** and EDX-maps **(d-g)** of ONWs@ZnPc RPAVD-O_2_-GLAD.

In this section, we will show the extension of the results above to a soft-template method for the fabrication of core@shell nanowires and nanotubes. Such a method has been developed during the last years for the synthesis of supported and on-device nanowires and nanotubes with applications as superhydrophobic and anti-freezing surfaces, nanoscale waveguides, nanosensors, excitonic solar cell photoelectrodes, and piezoelectric nanogenerators (Borras et al., 2009; Macias-Montero et al., 2013, 2017; Filippin et al., 2017b, 2019; Alcaire et al., 2019b). Scheme 2 presents the main steps for the fabrication of these hierarchical nanostructures. The foundation of the methodology is the formation of single-crystal organic nanowires by low-pressure vapor transport or physical vapor deposition on substrates previously decorated with nucleation centers. Metal and metal oxide thin films and nanoparticles deposited on a substrate as well as polymeric and organic surfaces have been demonstrated as suitable nucleation centers for the formation of small-molecule organic nanowires. In our previous articles (Borras et al., 2008, 2009, 2010; Macias-Montero et al., 2013, 2017; Filippin et al., 2017b, 2019; Alcaire et al., 2019b) we have optimized the process for Au, Ag, ITO, ZnO, TiO_2_, SiO_2_, PDMS, and organic substrates (Step i) and molecules as metal -porphyrins, -phthalocyanines and perylenes working as the nanowires building blocks (Step ii). Scheme 2 right shows SEM images of the different steps during the formation of the core@shell nanowires. Panel (b) shows a top-view image of the ONWs revealing squared morphology and extremely flat surfaces. The main parameters controlling the formation of organic nanowires from evaporable molecules are the substrate surface roughness and microstructure, temperature of the substrate in relation to the sublimation temperature of the molecules, pressure, growth rate and thickness (Borras et al., 2008, 2010). These NWs act as 1D or 3D (Borras et al., 2009; Filippin et al., 2017b) soft-template, compatible with vacuum and plasma deposition of layers, preferably when the deposition method yields the growth of conformal shells, as in the case of PECVD of inorganic and RPAVD of organic layers. Panel (c) in Scheme 2 shows a first insight of the RPAVD-O_2_ of a metal phthalocyanine molecule on as-grown organic nanowires. The result is the formation of a high density of hybrid core@shell nanowires with a shell morphology and composition ressembling those of the thin film counterpart. Figures 6a–d show SEM and TEM micrographs of several core@shell nanowires formed by RPAVD-O_2_. In general, this synthesis process leads to the decoration of the ONWs with radially distributed nanocolumns formed with similar aspects but smaller than those presented on flat substrates (see Figures 6b–d). The column width on ONWs is about 50% smaller than on a flat silicon substrate. The shell formation is usually conformal to the ONW acting as 1D support with nanocolumns radially distributed from the core (see Scheme 2c, Figures 6a–d) giving rise to highly conformal and hierarchical 3D nanostructures. However, shadowing effects are quite noticeable when working in glancing angle conditions (Figure 6e) or with long and tilted organic nanowires (Figures 6f,g). Thus, Figures 6e, 7 show the ZnPc RPAVD-O_2_-GLAD formation on the ONWs and demonstrate the difference in thicknesses between the nanocolumns formed on the side of the ONW facing toward the direction of the arriving precursor species. TEM (Figure 7a), HAADF-STEM (Figures 7b,c) micrographs, and EDX maps (Figures 7d–g) confirms the XPS and UV-Vis spectroscopy results discussed above on the formation of small ZnO particles under RPAVD-O_2_-GLAD conditions co-existing with ZnPc at the same time that reveals the porosity of the individual columns. The presence of a ZnO shell is revealed by EDX, while the inner part of the column seems to be mainly coming from the organic counterpart of the molecule.

**Figure 8:**
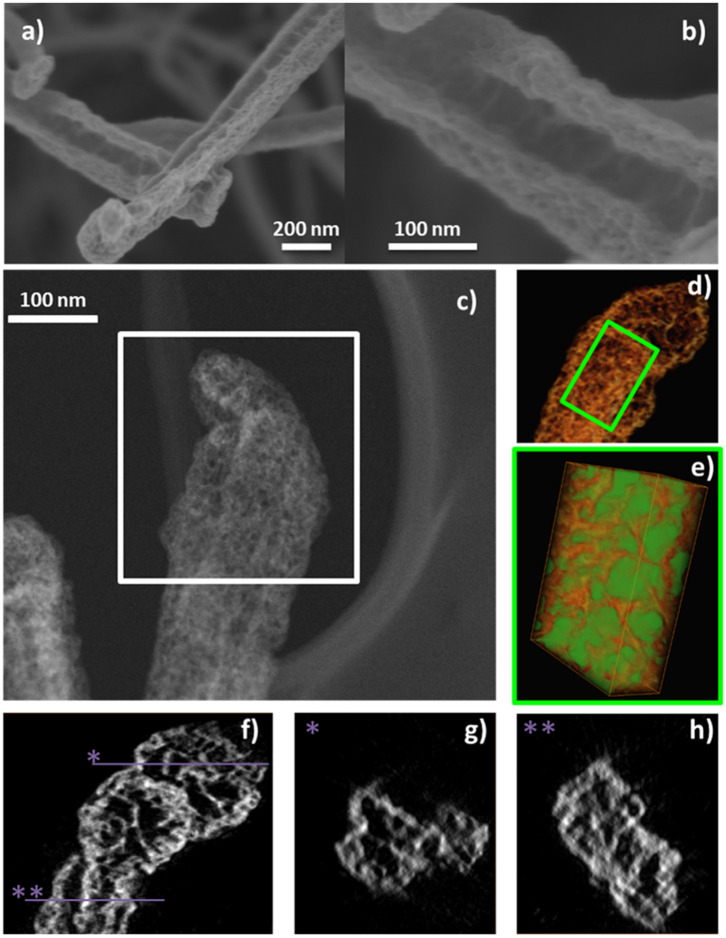
**(a,b)** SEM micrographs of an @TiClPc RPAVD-O_2_-SPE nanotube, **(c)** HAADF-STEM image at −60° of an isolated nanotube, selected for the electron tomography experiment. **(d)** Voxel rendering of the 3D reconstruction. **(e)** A subvolume was selected [inset in **(d)**] for the segmentation of the pores (displayed in green) and the porosity estimation. **(f)** Is an xy slice through the volume. **(g,h)** Are xz slices at the y positions indicated in **(f)**.

It is also interesting to address that in the previous examples the plasma conditions during the RPAVD-O_2_ process have been selected to keep part of the organic nanowire as the scaffold for the shell. Nevertheless, the organic core can be easily removed by annealing at mild temperatures during the formation of the shell or in a post-processing step as it is shown in the STEM micrograph in Figure 6d. The removal of the organic core leaves square or rectangular inner tubes with extremely flat walls (Filippin et al., 2017b; Macias-Montero et al., 2017). In addition, in concordance with the results regarding the thin film counterparts, once the hybrid core@shell nanowires are formed, we can apply the soft-plasma etching post-processing step in order to form metal or metal oxide shells. An example of the formation of highly porous TiO_2_ 1D nanostructures is presented in Figure 8. SEM images in panels (a,b) show the microstructure of the porous TiO_2_ shell formed around an empty channel left after the ONW removal. The HAADF-STEM (c) and 3D tomography reconstruction (d–h) micrographs provide detailed views of the porous systems, with a porosity estimation over the 44% and tiny pores with diameters between 2 and 20 nm. Panels (g,h) indeed demonstrate, in fair agreement with the results in Figure 5, that these pores are distributed all along the shell thickness, meaning that the plasma treatment affects the entire shell and not only the surface.

**Figure 9:**
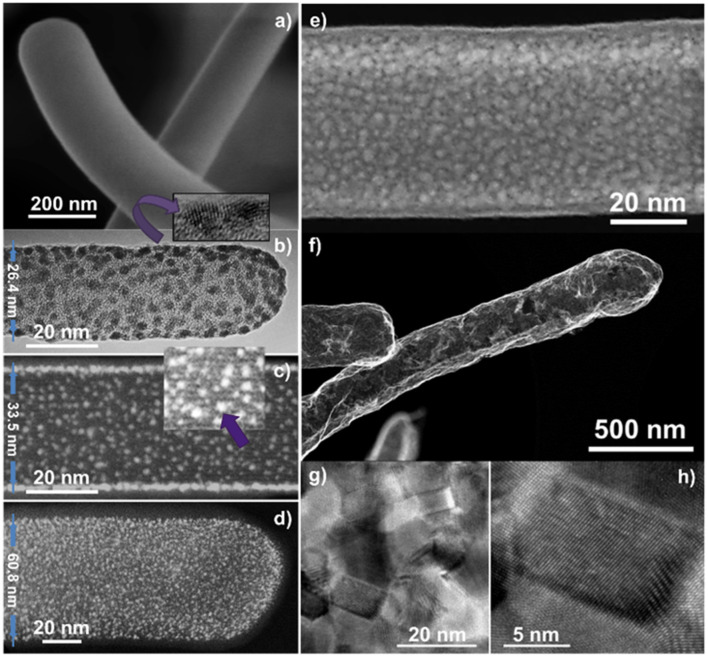
SEM **(a,f)**, TEM **(b)**, HAADF-STEM **(c–e)**, and High-Resolution TEM (inset in **b** and **g,h**) micrographs of PtOEP RPAVD-Ar shells on H_2_Pc nanowires **(a)** under different soft-plasma etching and annealing treatments.

In addition to the formation of metal oxide porous layers and shells, this methodology can be applied for the growth of metal nanostructures. Figure 9 gathers characteristic SEM, TEM, and STEM micrographs of the procedure applied to the formation of metal Pt nanoparticles. Panel (a) demonstrates that the PtOEP RPAVD-Ar shells are rounded at the tip and cylindrical along the axis, losing the squared shape of the H_2_Pc core. As previously settled, the RPAVD-Ar process yields the formation of PtOEP composite layers where the PtOEP molecules appear embedded in a polymer-like matrix (Filippin et al., 2017a). Once the Pt precursor layer is deposited, the core@shell nanowires are exposed to a soft plasma etching treatment. Depending on the thickness of the PtOEP layer and the conditions of the plasma etching, the post-treatment may be adjusted to be soft enough so as to yield tiny NPs. With only 30 min of treatment at room temperature, NP decorated NWs were achieved as observed in Figure 9b. High-resolution TEM reveals the crystalline nature of the Pt NPs formed (inset in panel b), whilst carefully focusing the core, the original H_2_Pc molecular planes are still observable (Figure 9c), proving that this soft etching condition has not compromised the integrity of the inner core. This is one of the main advantages of the RPAVD methodology. The Pt NPs had a mean diameter of 1.5 ± 0.2 nm according to Figures 9c,d. The NPs in (b) presents a mean diameter of 2.2 ± 0.4 nm, noticeable bigger than in (c,d). This difference results from the smaller diameter of the NW in (b), where adjacent NPs joint more easily into bigger ones. When the etching process was prolonged to 60 min, i.e., twice the time for generating NPs, a semi-percolated Pt shell was obtained (Figure 9e). Highly percolated platinum shells were also obtained by increasing the thickness of the PtOEP shell and by implementing a more severe etching treatment. It must be mentioned that the formation of nanoparticles was carried out at relatively low plasma power (300 W), room temperature and short treatment time (1-h maximum), while the formation of a platinum layer requires higher power (600 W), temperature (180°C), and prolonged treatment time. The obtained platinum nanoparticles or shell are crystalline as confirmed by HRTEM in Figures 9g,h.

## Conclusions

We have developed a general protocol for the formation of hybrid and porous metal oxides by a vacuum process in combination with oxygen plasma etching by extending the remote plasma assisted method to the use of commercially available metal phthalocyanines and porphyrins. We have demonstrated the versatility of the method from the point of view of the chemical composition of the porous metal and metal oxide layers through the fabrication of ZnO, CuO, Fe_2_O_3_, TiO_2_, PtO, and Pt porous layers. We have shown as well that a plethora of microstructures as continuous and homogeneous layers, nanocolumns and nanoparticles can be achieved by tuning the plasma experimental conditions as gas composition (Ar or Ar + O_2_), layer thicknesses and soft-plasma etching duration and substrate temperature. The deposition under glancing angle conditions provides the formation of tilted nanocolumns showing high inner porosity. The protocol has been also extended to the development of core@shell nanowires and nanotubes using supported organic nanowires as 1D templates. The combination of SEM and advanced 3D characterization has provided the full elucidation of the microstructure of the sample. Particularly, FIB-3D and electron tomography have been used to reveal the porosity of these low dimensional systems showing nanopores distributed through the complete thickness of the metal oxide thin films and shells. HAADF-STEM micrographs have also proved that the methodology can be compatible with the deposition on sensitive surfaces, such as the organic nanowires themselves. These results demonstrate that the combined RPAVD and soft-plasma etching procedure is a striking methodology for the development of highly porous systems supported on substrates or on-device architectures.

## Experimental Section

### Metal Complex and Organic Precursors

Phthalocyanine (H_2_Pc), cobalt-phthalocyanine (CoPc), Zinc phthalocyanine (ZnPc), copper phthalocyanine (CuPc), fluorinated copper phthalocyanine (F16CuPc), chlorine iron phthalocyanine (ClFePc), chlorine titanium phthalocyanine (ClTiPc) from Sigma-Aldrich, Platinum octaethylporphyrin (PtOEP) from Frontiers and perylene bisimide (MePTCDI) from Sensient Imaging Technologies, were used as received.

### Synthesis of Oxide Metal-Phthalocyanine and Metal-Porphyrins Thin Films by RPAVD-Ar and RPAVD-O_2_

An schematic of the plasma reactor is shown in Figure S1. The base pressure of the reactor was 10^−6^ mbar. The molecules were sublimated using the Knudsen cell (at about 10 cm from the substrates) under Ar atmosphere (SUBL), Ar plasma (RPAVD-Ar) and Ar + O_2_ or O_2_ (RPAVD-O_2_) plasma in the downstream region of a MW-ECR reactor. The samples were deposited under two geometries into the deposition chamber, named normal deposition and glancing angle (GLAD). Gas pressure was dosed by a calibrated mass flow controller being 3·10^−2^ mbar for samples fabricated under normal deposition conditions, and 1·10^−3^ mbar in GLAD. The oxide metal-phthalocyanine thin films were deposited simultaneously on the ONWs, described in the previous section, silicon Si(100) and fused silica substrates. The substrates were at RT, and placed facing down to the Knudsen cell and facing away to the plasma discharge. Table 1 presents the working conditions in the fabrication process of the samples appearing throughout this work. The ONWs and the metal oxide films oxide thin films working as core and shell in the hierarchical NWs, respectively, are both fabricated in the same deposition reactor.

### Soft Oxygen Plasma Etching (SPE) Treatments by ECR-MW

The post-treatment of the samples by soft plasma etching was produced in the same reactor with the samples facing down the plasma glow discharge. The distance between the substrates and the glow discharge region was fixed to 8 cm. Several SPE experimental conditions were tested to complete the formation of the metal oxide porous layers with pure O_2_ and Ar + O_2_ plasma gases, power in the range between 300 and 600 W, substrate temperatures from RT to 180°C, and treatment durations from several minutes to 140 min.

### Deposition of Nucleation Centers

Two types of nucleation centers were used as seeds for the NWs growing process: Ag nanoparticles and oxide thin films. The Ag nanoparticles by sputtering DC were fabricated using a silver wire operating at bias of 450 V under an argon flow of 10^−4^ mbar (Macias-Montero et al., 2015). TiO_2_, ZnO, and SiO_2_ thin films were synthesized by plasma enhanced chemical vapor deposition (PECVD) under the conditions defined elsewhere (Borrás et al., 2006; Borras et al., 2007b; Romero-Gómez et al., 2010). The precursors used were Titanium tetra-isopropoxide (TTIP), Diethyl Zinc (DEZ), and Chlorotrimethyl silane (CITMS) for the production of TiO_2_, ZnO, and SiO_2_, respectively. The plasma was produced in a microwave electron cyclotron resonance configuration (MW-ECR) at 400 and 600 W (Borras et al., 2007a,b; Alcaire et al., 2019b; Filippin et al., 2019). The substrates were at RT.

### Synthesis of ONWs by PVD

The sublimation of the molecule was carried out using a Knudsen cell, placed at 8 cm from the substrates, under 10^−2^ mbar of Ar flow, which was dosed by a calibrated mass flow controller (Figure S1). Growth rate and equivalent thickness of the CoPc and Perylene NWs were monitored using a quartz crystal microbalance (QCM), and the growth rate adjusted to 0.3–0.4 Å/s using a density of 0.5 g/cm^3^ in the QCM electronic. The substrate temperature was imposed at 170°C for H_2_Pc, 180°C for CoPc, and 140°C for MePTCDI in a heateable sample holder connected to an electric current source, in order to induce the formation of the organic nanowires.

### Experimental Characterization Methods

High-resolution SEM images of the samples deposited on silicon wafers were obtained in a Hitachi S4800 microscope at an acceleration voltage of 2 kV. Cross sectional views were obtained by cleaving the Si (100) substrates. FIB-3D was performed on a Zeiss crossbeam 550 FIB-SEM. TEM images were obtained in a CM20 apparatus from Philips. HAADF-STEM images were acquired in a Tecnai G2 F30 S-Twin STEM and a Titan Themis from Thermo Fisher Scientific (formerly FEI). Electron tomography was performed in a Titan Themis using a Fischione tomography holder. XPS experiments were performed in a Phoibos 100 DLD x-ray spectrometer from SPECS. The spectra were collected in the pass energy constant mode at a value of 50 eV using magnesium and aluminum X-ray sources. C1s signal at 284.5 eV was utilized for calibration of the binding energy (BE) in the spectra. The assignment of the BE to the different elements in the spectra corresponds to the data in Briseno et al. (2008b). UV-Vis transmission spectra of samples deposited on fused silica slides were recorded in a Cary 100 spectrophotometer in the range from 190 to 900 nm.

## Data Availability Statement

The datasets generated for this study are available on request to the corresponding author.

## Author Contributions

JO, MA, AF, and MM-M produced the samples in thin film and nanowires with assistance of JS-V and FA. JS-V and FA were in charge also of transmittance UV-Vis spectroscopy. JE carried out the XPS analyses. TR and ZS performed the HREM, HAADF-STEM, and EDX analysis. CM performed the FIB-3D experiment. MJ and ZS worked together in the electron tomography experiment. ABa and ABo designed the experiments. All authors contributed to the article and approved the submitted version.

## Conflict of Interest

The authors declare that the research was conducted in the absence of any commercial or financial relationships that could be construed as a potential conflict of interest.

## Supplementary Material

Supplementary Material includes additional XPS results and a schematic of the plasma-vacuum reactor.
