# Supplementary material for: Supported Porous Nanostructures Developed by Plasma Processing of Metal Phthalocyanines and Porphyrins
Source: Front Chem. 2020 Jun 17;8:520. doi: 10.3389/fchem.2020.00520 (PMC7311806; doi:10.3389/fchem.2020.00520)

Supplementary Material

Tables S1. Atomic percentage estimated by XPS of representative samples.

Sample \ % atom	Zn %	N %	C %	O %
ZnPc SUBL	4.25	24.2	70.35	1.2
ZnPc RPAVD-O_2	3.7	14.5	49.1	32.7
ZnPc RPAVD-O_2 GLAD	26.5	4.2	39.3	30.0
ZnPc RPAVD-O_2 + SPE	4.7	14.9	51.1	29.3
ZnPc RPAVD-O_2 + SPE (prolonged)	16.6	7.8	44.1	31.5
ZnPc RPAVD-O_2 GLAD + SPE (prolonged)	23.9	8.9	31.0	36.2

Sample \ % atom	Cu %	N %	C %	O %
CuPc form (C32H16CuN8)	2.4	19.6	78	-
CuPc SUBL 200ºC + SPE 10 min	3.0	6.5	71.3	19.2
CuPc SUBL 200ºC + SPE 40 min	5.1	4.8	54.1	36.0
CuPc RPAVD-O_2	2.9	13.4	56.8	26.9
CuPc RPAVD-O_2 + SPE (prolonged)	17.0	9.0	38.9	35.1

Sample \ % atom	Cu %	N %	C %	O %	F %
F_{16}CuPc SUBL	1.5	10.5	57.8	1.2	29.0
F_{16}CuPc RPAVD-O_2	3.0	11.0	46.5	21.5	18.0
CuPc RPAVD-O_2 + SPE	4.6	12.75	56.0	26.65	-
CuPc RPAVD-O_2 + SPE (prolonged)	17.0	9.0	38.9	35.1	-

Sample \ % atom	Fe %	N %	C %	O %	Cl %
ClFePc RPAVD-O_2	22.4	21.9	38.0	16.1	1.7

Sample \ % atom	Ti %	N %	C %	O %	Cl %
ClTiPc SUBL	2.17	15.4	77.82	4.18	0.43
ClTiPc RPAVD-O_2 + SPE	26.8	-	12.2	59.3	0.47

Sample \ % atom	Pt(0) %	Pt (PtOEP) %	Pt (PtO-PtO2) %	N %	C %	O %	Si %
PtOEP Sublimated	-	2.9	-	6.7	85.5	4.9	-
PtOEP RPAVD-O_2	-	5.8	7.6	1.7	27.9	47.6	9.4
PtOEP RPAVD-Ar	-	2.4	-	7.1	71.7	18.8	-
PtOEP RPAVD-O_2 + SPE+ Anneal (H_2+Ar)	11.1	-	-	1.5	30.7	35.1	21.6

Figure S1. Experimental set-up

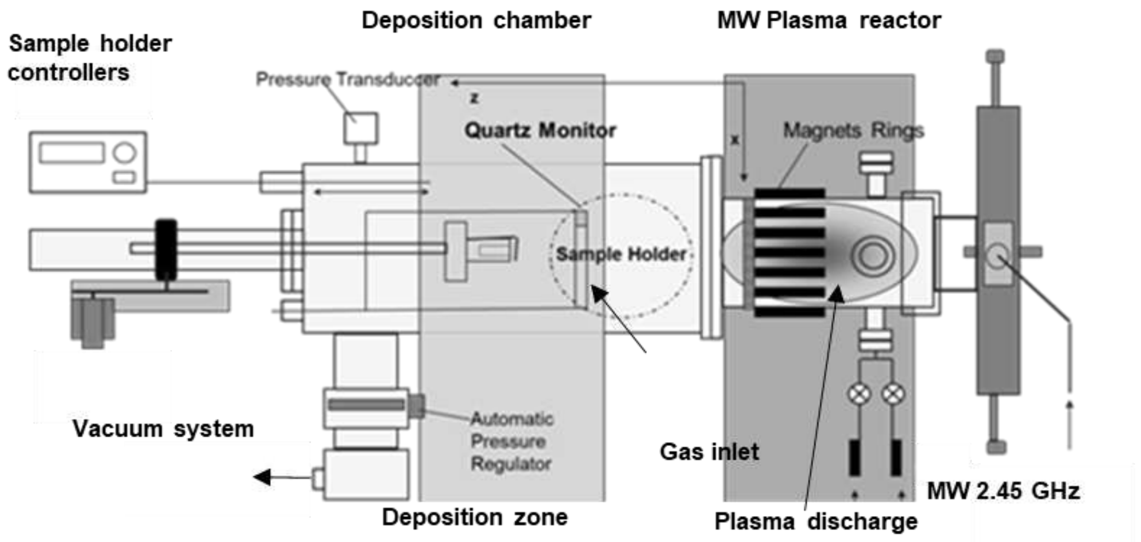

Supplement: Supplementary file 1 [file Data_Sheet_1.pdf]
